# The association between neonatal respiratory distress syndrome and gut microbiota dysbiosis: evidence from metagenomics

**DOI:** 10.3389/fmicb.2026.1925400

**Published:** 2026-09-01

**Authors:** Faqun Liu, Jinghua Luo, Lihua Feng, Yanan Xia, Xiangxiang Chen, Yunping Xu, Yang Liu

**Affiliations:** Department of Pediatrics, The Second Affiliated Hospital, Jiangxi Medical College, Nanchang University, Nanchang, China

**Keywords:** gut microbiota, gut-lung axis, metagenomics, neonatal respiratory distress syndrome, preterm neonates

## Abstract

**Objective:**

Neonatal respiratory distress syndrome (NRDS) is a common and life-threatening respiratory disorder that affects newborns, significantly impacting their health and survival rates. In recent years, the potential role of the gut-lung axis in NRDS has garnered increasing attention; however, the specific mechanisms involved remain unclear. This study aimed to investigate the association between NRDS and gut microbial composition and functional profiles using shotgun metagenomic sequencing.

**Methods:**

This case–control study recruited 25 infants diagnosed with NRDS and 15 healthy newborns, all aged between 2 and 6 days. Fecal samples were collected from all participants for subsequent metagenomic sequencing and bioinformatics analysis.

**Results:**

There was no significant difference in α-diversity between the NRDS group and the control (CON) group (*p* = 0.3335). However, β-diversity exhibited a significant difference (*p* = 0.001). In the NRDS group, the gut microbiota was enriched with Bacillota, *Klebsiella*, and *Enterococcus*, while *Bifidobacterium* and butyrate metabolism-related bacteria (*Bacteroides*/Butyricicoccus) were significantly reduced (*p* < 0.05). Functional analysis indicated that *Staphylococcus aureus* infection and the Phosphotransferase system (PTS) pathway were enriched in the NRDS group, whereas Butanoate metabolism and Glutathione metabolism were significantly enriched in the CON group. A 10-species classifier achieved 96% AUC for NRDS prediction.

**Conclusion:**

NRDS was associated with differences in gut microbial composition and shotgun-metagenomic functional profiles. These preliminary findings characterize group-level microbial differences but do not establish whether the observed alterations preceded or resulted from NRDS. Longitudinal studies are required to clarify their temporal relationship and clinical relevance.

## 1 Introduction

Neonatal respiratory distress syndrome (NRDS) is a common and serious condition that affects the breathing of newborns, which poses significant risk to their lives (Marseglia et al., 2019). The primary pathophysiological mechanism involves the synthesis, secretion, or dysfunction of pulmonary surfactant (PS). This dysfunction results in alveolar collapse and decreased lung compliance. NRDS is clinically characterized by the onset and progressive worsening of respiratory distress shortly after birth (Chi et al., 2018). Recent multicenter cohort research has indicated that mortality rates for NRDS can reach as high as 17–24%, establishing it as a major factor contributing to neonatal mortality and disability (De Luca et al., 2022). Despite progress in clinical therapies, the frequency of this condition continues to increase each year. The fundamental pathological processes are not fully understood, and the condition is linked to prolonged health complications, such as chronic lung disease and neurodevelopmental disorders (Wang et al., 2023). It is essential to gain insights into the mechanisms driving NRDS, improve diagnostic tools and identify therapeutic targets.

The gut microbiota plays a significant role in human immunity, metabolism, and neural development (Cryan et al., 2019). Recent studies have shown that microbial communities are closely related to various respiratory diseases (Natalini et al., 2023). Damage to lung tissue can modify the composition of lung microbial communities, amplify inflammation, hinder immune defenses, and initiate a feedback loop that exacerbates acute lung injury (Zhang et al., 2022). The gastrointestinal tract, which is the largest reservoir of microbes, plays a crucial role in influencing lung diseases via the “gut-lung axis” (He et al., 2017). In contrast to older children and adults, newborns display unique structures of microbial communities, which are affected by elements such as gestational timing and mode of delivery (Pattaroni et al., 2018). Consequently, it is crucial to explore the impact of the neonatal lung-gut microbiota on the progression of NRDS.

We conducted a shotgun metagenomic analysis to characterize differences in gut microbial composition and functional profiles between neonates with NRDS and healthy controls. This exploratory study aimed to identify NRDS-associated microbial features without inferring a causal relationship.

## 2 Materials and methods

### 2.1 Subjects

This study was approved by the Biomedical Research Ethics Committee of the Second Affiliated Hospital of Nanchang University (Approval No. O - Medical Research Ethics Review [2024] No. (90); review date: 27 August 2024; expedited review, initial review). Written informed consent (version V3.0, dated 21 August 2024) was obtained from the parents or legal guardians of all participating neonates prior to enrollment. All procedures were performed in accordance with the Declaration of Helsinki and the relevant institutional guidelines. From November 2024 to August 2025, 25 neonates with NRDS (diagnosed per PALICC-2 criteria (Emeriaud et al., 2023)) and 15 healthy controls (CON) were enrolled. Inclusion criteria for NRDS: (1) Complete clinical data; (2) Parental consent. Exclusion criteria: (1) Severe infection/sepsis; (2) Congenital/genetic disorders; (3) Tuberculosis; (4) Incomplete data; (5) Guardian refusal. CON comprised healthy infants undergoing routine checkups. Maternal and neonatal demographic data were collected.

### 2.2 Sample collection and DNA extraction

Fresh stool specimens (1–2 g) were collected with sterile swabs immediately after neonatal defecation, transferred to 2 mL EP tubes, and stored at −80 °C until DNA extraction. Genomic DNA was isolated from 0.5 g aliquots using the FastPure Stool DNA Isolation Kit (Magnetic bead, MJYH, China) according to manufacturer protocols. DNA concentration and purity were quantified via Synergy HTX and NanoDrop 2000, respectively, followed by 1% agarose gel electrophoresis for quality validation.

### 2.3 Metagenomic sequencing

The DNA extract was processed to reach an approximate length of 400 base pairs using the Covaris M220 (Gene Company Limited, China) for creating a paired-end library. The library was developed with the help of NEXTFLEX Rapid DNA-Seq (Bioo Scientific, Austin, TX, USA). Sequencing of the end pairs was performed on the Illumina NovaSeq™ X Plus (Illumina Inc., San Diego, CA, USA) at Majorbio Bio-Pharm Technology Co., Ltd. (Shanghai, China), in accordance with the recommendations of the manufacturer for the NovaSeq X Series 25B Reagent Kit^1^.

### 2.4 Processing of metagenome sequencing data

Raw sequencing data were analyzed on the Majorbio Cloud Platform. Adapters and low-quality reads (length < 50 bp, Phred score < 20, or containing N bases) were filtered using fastp. Human-derived sequences were removed via BWA alignment. *De novo* assembly was performed with MEGAHIT (contig length cutoff: 300 bp). ORFs (≥100 bp) were predicted using Prodigal, followed by CD-HIT clustering at 90% identity/coverage to generate a non-redundant gene catalog. Gene abundance quantification was conducted with SOAPaligner at 95% identity (Li et al., 2008).

### 2.5 Taxonomic and functional annotation

Non-redundant genes were classified by DIAMOND (Buchfink et al., 2015) alignment (e-value < 1e−5) against the NCBI NR database. Functional annotations were integrated with taxonomic profiles for multi-level differential analysis (taxonomic, functional, and gene-level) using the Kruskal-Wallis test.

### 2.6 Statistical analysis

Data were analyzed using SPSS 26.0 and Majorbio Cloud Platform^2^. Quantitative variables underwent normality (Shapiro–Wilk) and homogeneity of variance (Levene’s) tests. Normally distributed data were expressed as mean±SD (t-test), non-normal as median [IQR] (Mann–Whitney U test). Categorical data were presented as frequency (%) (*χ*^2^ test). Significance threshold was *p* < 0.05.

## 3 Results

### 3.1 Basic characteristics of the NRDS and CON

A total of 40 preterm neonates aged 2–6 days were enrolled, including 25 in the NRDS group and 15 in the control (CON) group. No statistically significant between-group differences were observed in sex, gestational age, postnatal age at sampling, mode of delivery, feeding method, stool characteristics, or maternal age (*p* > 0.05; Table 1). All stool samples were yellow, consistent with transitional or mature stool. All neonates initiated enteral feeding within 24 h after birth, and none received antibiotics before stool collection. All neonates in the NRDS group received exogenous pulmonary surfactant (porcine pulmonary phospholipids) within 6 h after birth, and stool samples were collected at least 48 h after administration. No neonates in the CON group received pulmonary surfactant.

**Table 1:** Characteristics of the study subjects.

Characteristics	NRDS group	CON group	*t*/*Z*/*χ*^2^	*p*
	*N* = 25	*N* = 15		
Female (*n*, %)	13 (52.00)	7 (46.70)	0.107	0.744
Gestational age (days)	250.24 ± 7.54	254.60 ± 7.02	−1.836	0.074
Age at sampling (days)	4.00 (3.00, 5.00)	5.00 (3.00, 5.00)	−0.923	0.356
Stool type, (Yellow transitional/mature, *n*, %)	25 (100)	15 (100)	—	—
Meconium, (*n*, %)	0 (0)	0 (0)	—	—
Birth weight (kg)	2.29 ± 0.36	2.49 ± 0.46	−1.385	0.173
Enteral feeding within 24 h of birth (*n*, %)	25 (100)	15 (100)	—	—
Antibiotic exposure before sampling (*n*, %)	0 (0)	0 (0)	—	—
Surfactant administration (*n*, %)	25 (100)	0 (0)	—	<0.001
Vaginal birth (*n*, %)	8 (32.00)	6 (40.00)	0.264	0.608
Mixed feeding (*n*, %)	15 (60.00)	10 (66.67)	0.178	0.673
soft stool (*n*, %)	19 (76.00)	13 (86.70)	−0.923	0.615
Mother’s age (years)	30.72 ± 5.23	29.00 ± 4.84	1.034	0.307

Data: mean ± SD or median [IQR]; Intergroup comparisons: *t*-test/Mann–Whitney U test for continuous variables, *χ*^2^ test for categorical variables.

### 3.2 Microbial diversity and composition

A metagenomic approach was utilized to investigate the microbial communities and compositions present in the NRDS and CON groups. Following quality control filtering, 98–99% of the sequences were classified as high-quality. The ACE index for the NRDS group was significantly lower than that of the CON group, indicating that the CON group possessed a greater total number of species. The α-diversity indices, including the Shannon and Simpson, demonstrated relatively comparable values between the two groups (Table 2). This finding suggests that, although there has been a shift in the overall species composition of intestinal microorganisms within the NRDS group, the alterations in community structure remain minimal.

**Table 2:** α diversity analysis of intestinal microbe and metagenomic information statistics represent the significance of differences in different samples.

Samples		NRDS group	CON group	t/Z	*p*
		*N* = 25	*N* = 15		
ACE	Total	507.00 (210.30, 652.00)	620.00 (388.00, 735.00)	−1.241	0.214
	Bacteria	471.00 (191.00, 537.00)	550.00 (352.00, 693.00)	−1.508	0.132
	Fungi	0.21 ± 0.59	0.27 ± 0.46	−0.327	0.746
	Viruses	61.46 ± 54.50	46.33 ± 36.25	1.040	0.305
	Eukaryota	3.48 ± 2.76	3.07 ± 3.06	0.441	0.662
	Archaea	0.13 ± 0.34	0.07 ± 0.26	0.571	0.571
Shannon	Total	1.76 (1.40, 2.10)	1.88 (1.50, 2.40)	−0.981	0.326
	Bacteria	1.64 (1.40, 2.10)	1.86 (1.40, 2.30)	−1.030	0.303
	Fungi	0.00 (0.00, 0.00)	0.00 (0.00, 0.00)	−1.133	0.257
	Viruses	1.87 (0.90, 3.00)	0.93 (0.70, 1.30)	−2.309	0.021*
	Eukaryota	0.48 (0.20, 0.90)	1.02 (0.60, 1.30)	−2.836	0.005**
Simpson	Total	0.29 (2.00, 4.00)	0.25 (0.20, 0.40)	−1.184	0.237
	Bacteria	0.31 (0.20, 0.40)	0.28 (0.20, 0.40)	−1.060	0.289
	Fungi	0.10 ± 0.27	0.40 ± 0.51	−2.133	0.046*
	Viruses	0.34 (0.10, 0.50)	0.54 (0.40, 0.70)	−2.309	0.021*
	Eukaryota	0.72 (0.50, 0.90)	0.45 (0.30, 0.70)	−2.371	0.005**
	Archaea	0.33 ± 0.48	0.13 ± 0.35	1.494	0.144
Sequence information	Clean reads	43875714.00 (42914441.00, 45311492.00)	43656982.00 (42481660.00, 44009956.00)	−1.187	0.235
	Clean base(bp)	6605852969.00 (6454467809.50, 6826353626.00)	6575541948.00 (6400291465.00, 6631072261.00)	−1.020	0.308
	Percent in raw reads (%)	99.30 (99.20, 99.40)	99.37 (99.30, 99.50)	−1.215	0.224
	Percent in raw bases (%)	99.03 (98.80, 99.20)	99.16 (98.90, 99.20)	−1.718	0.086

Data: mean ± SD or median [IQR]; Intergroup comparisons: *t*-test/Mann–Whitney U test for continuous variables, *χ*^2^ test for categorical variables; *p < 0.05, **p < 0.01.

**Figure 1:**
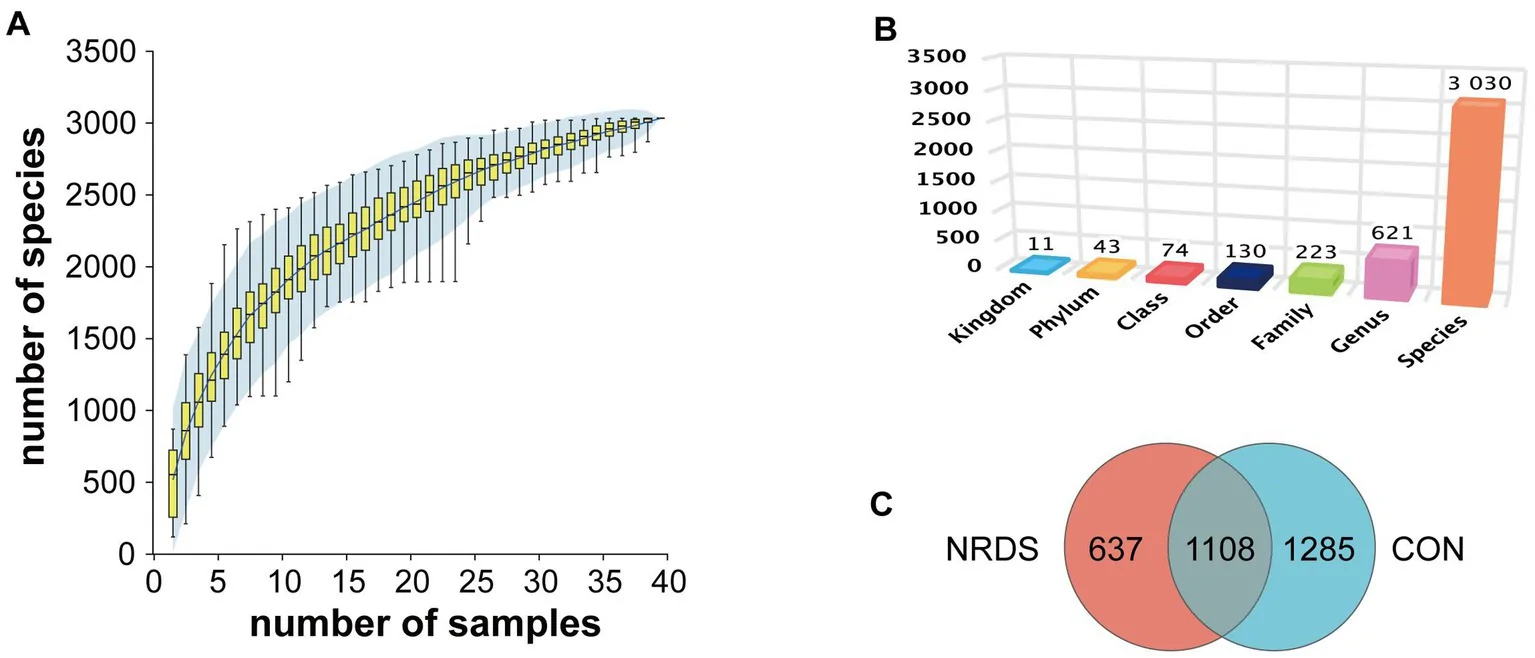
Microbial community composition and difference. **(A)** Species accumulation curve. **(B)** Species statistical chart. **(C)** The Venn diagram shows the number of shared and unique species.

This study revealed that the species accumulation curve tends to flatten, suggesting that an increase in sample size will not yield a significant number of new species and that the current sample size is adequate for analysis (Figure 1A). The two sample groups collectively encompassed eight major taxonomic levels, ranging from domain to species (Figure 1B). The NRDS group exhibited significantly fewer unique species (637) compared to the CON group (1,285), indicating a reduction in gut microbiota diversity and an ecological imbalance in NRDS infants. The proportion of shared species between the two groups was only approximately 37% (1,108/3030), highlighting the specific alterations in microbiota structure under disease conditions (Figure 1C).

**Figure 2:**
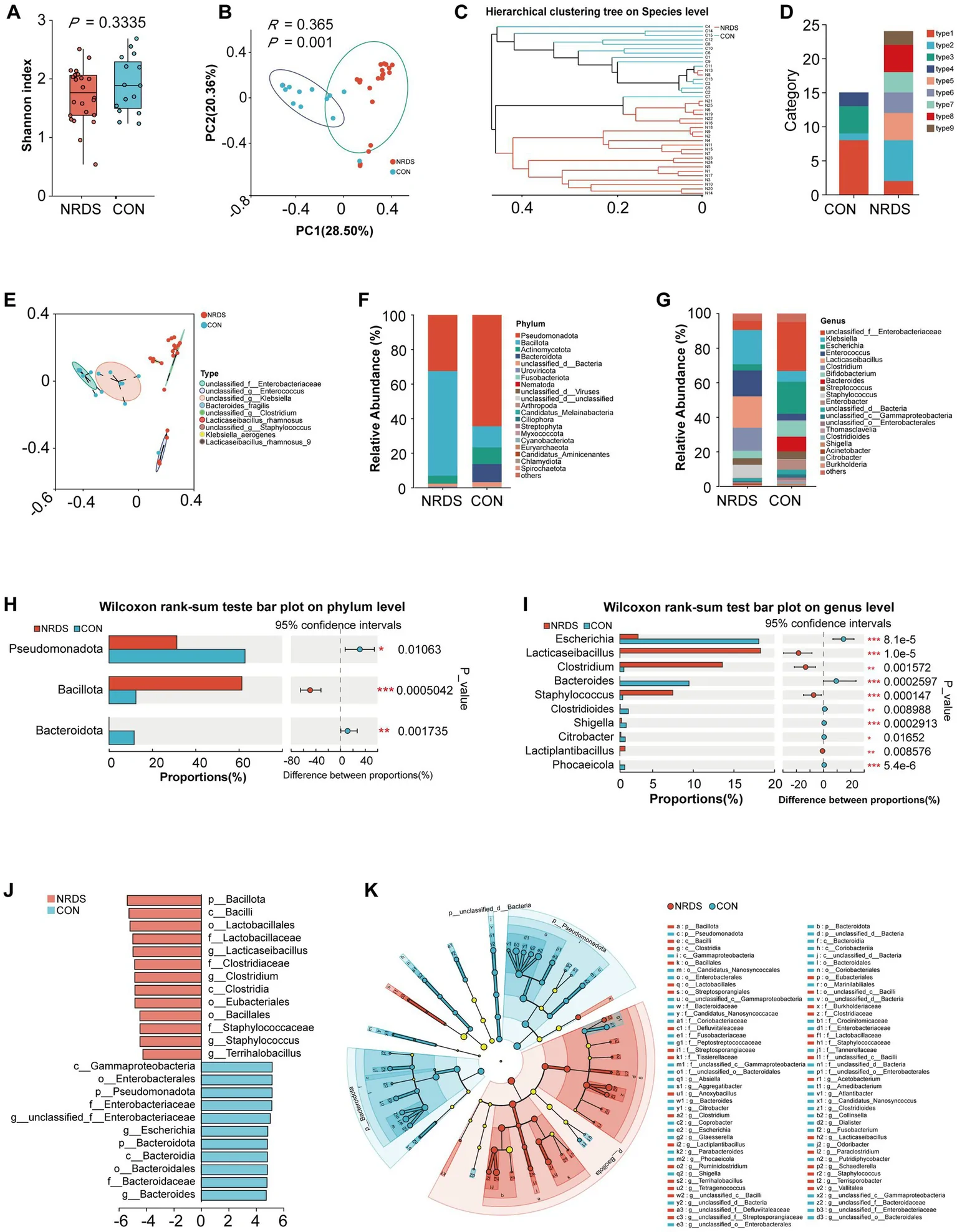
Gut microbiota diversity and taxonomic differences between NRDS and CON neonates. **(A,B)** α-Diversity and β-diversity. **(C)** Hierarchical clustering of microbial communities. **(D,E)** Enterotype distribution and dominant species. **(F,G)** Phylum- and genus-level composition. **(H,I)** Differentially abundant taxa (*p ≤ 0.05, **p ≤ 0.01, ***p ≤ 0.001). **(J)** LEfSe analysis of discriminative features (LDA > 4). **(K)** Phylogenetic tree (LDA > 3). PCoA, Principal coordinates analysis; LEfSe, LDA effect size analysis; LDA, Linear discriminant analysis.

β-diversity analysis (PERMANOVA, *p* = 0.001, *R* = 0.365) revealed significant structural differences between NRDS and CON groups, despite comparable α-diversity (Shannon index, *p* = 0.334, Figures 2A,B). Hierarchical clustering demonstrated distinct microbial colonization patterns (Figure 2C), with enterotype analysis showing NRDS-enriched type 2 dominated by *Klebsiella*, contrasting with CON-predominant type 1 featuring *Bacteroides* (Figures 2D,E).

At the phylum level, NRDS exhibited elevated Bacillota (formerly Firmicutes) and reduced *Pseudomonadota* (formerly *Proteobacteria*), *Bacteroidota* (formerly *Bacteroidetes*), and *Actinomycetota* (formerly *Actinobacteria*) (*p* < 0.05, Figures 2F,H). Genus-level profiling identified increased *Klebsiella*, *Enterococcus*, and *Staphylococcus* alongside decreased *Bifidobacterium* and *Bacteroides* in NRDS (*p* < 0.05, Figures 2G,I). LEfSe analysis (LDA > 4) confirmed differential enrichment of Bacillota lineages in NRDS versus Bacteroidota/Pseudomonadota in CON (Figures 2J,K).

### 3.3 Functional profiling of gut microbiota

**Figure 3:**
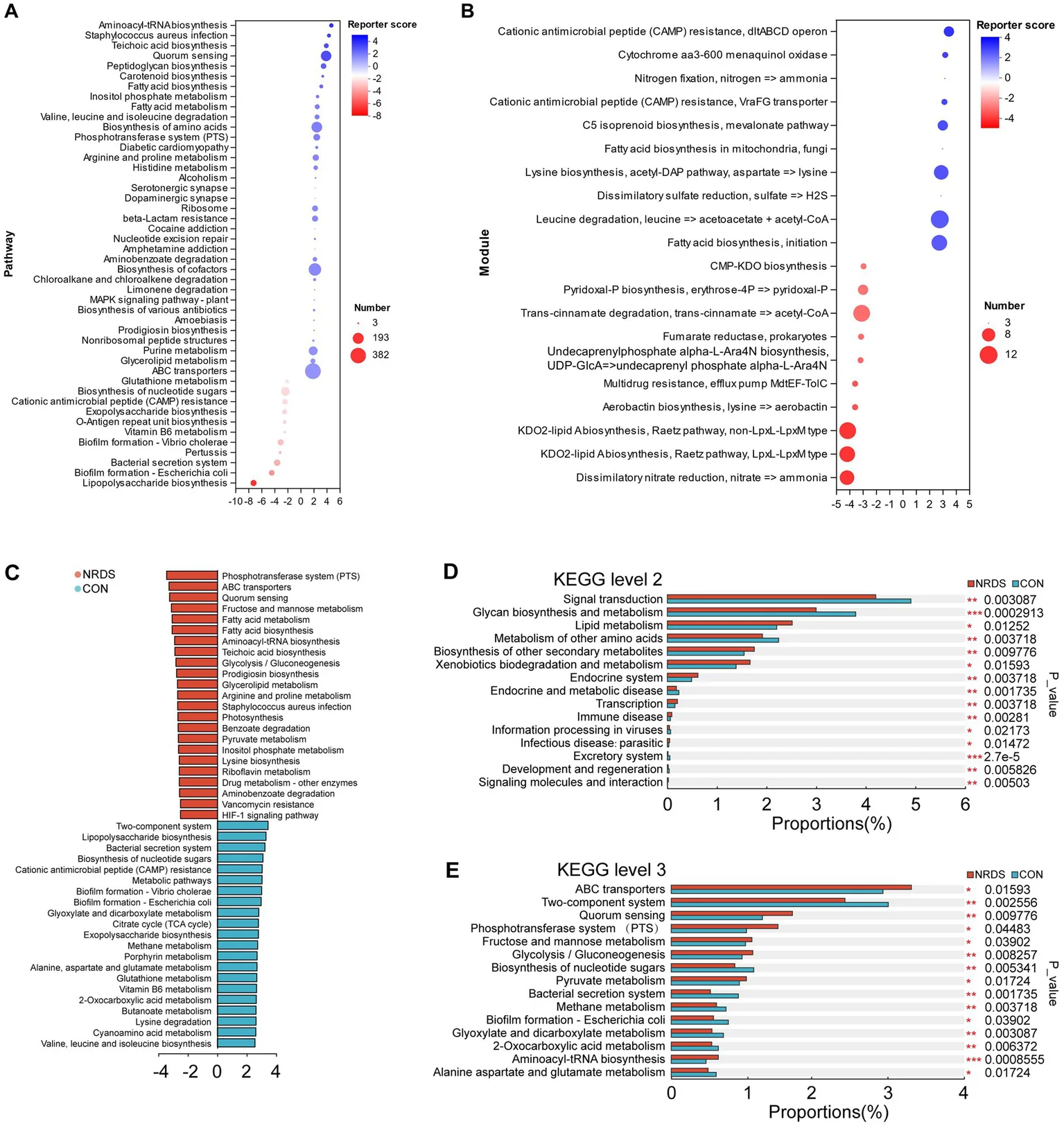
Functional differences in gut microbiota between NRDS and CON neonates. **(A,B)** Heatmaps of KEGG pathway and module enrichment (|reporter score| > 1.65). Blue: NRDS-enriched; Red: CON-enriched. Bubble size indicates gene count. **(C)** Differential pathway analysis at KEGG level 3. **(D,E)** Predicted metagenomic differences (*p ≤ 0.05, **p ≤ 0.01, ***p ≤ 0.001). KEGG, Kyoto Encyclopedia of Genes and Genomes; KO, KEGG Orthology.

This study identified a total of 386 KEGG pathways and 361 KEGG modules, of which 59 KEGG pathways (43 enriched in NRDS and 16 enriched in CON) and 65 KEGG modules (39 enriched in NRDS and 26 enriched in CON) exhibited significant differences in enrichment (Figures 3A,B). In the NRDS group, significant enrichment was observed in pathways related to *Staphylococcus aureus* infection, the phosphotransferase system (PTS), the PPAR signaling pathway, and the Toll-like receptor signaling pathway. In contrast, the CON group displayed significant enrichment in pathways associated with glutathione metabolism, vitamin B6 metabolism, and butyrate metabolism (Figures 3C–E).

### 3.4 The connection between species and function

#### 3.4.1 Butyrate metabolism impairment in NRDS

**Figure 4:**
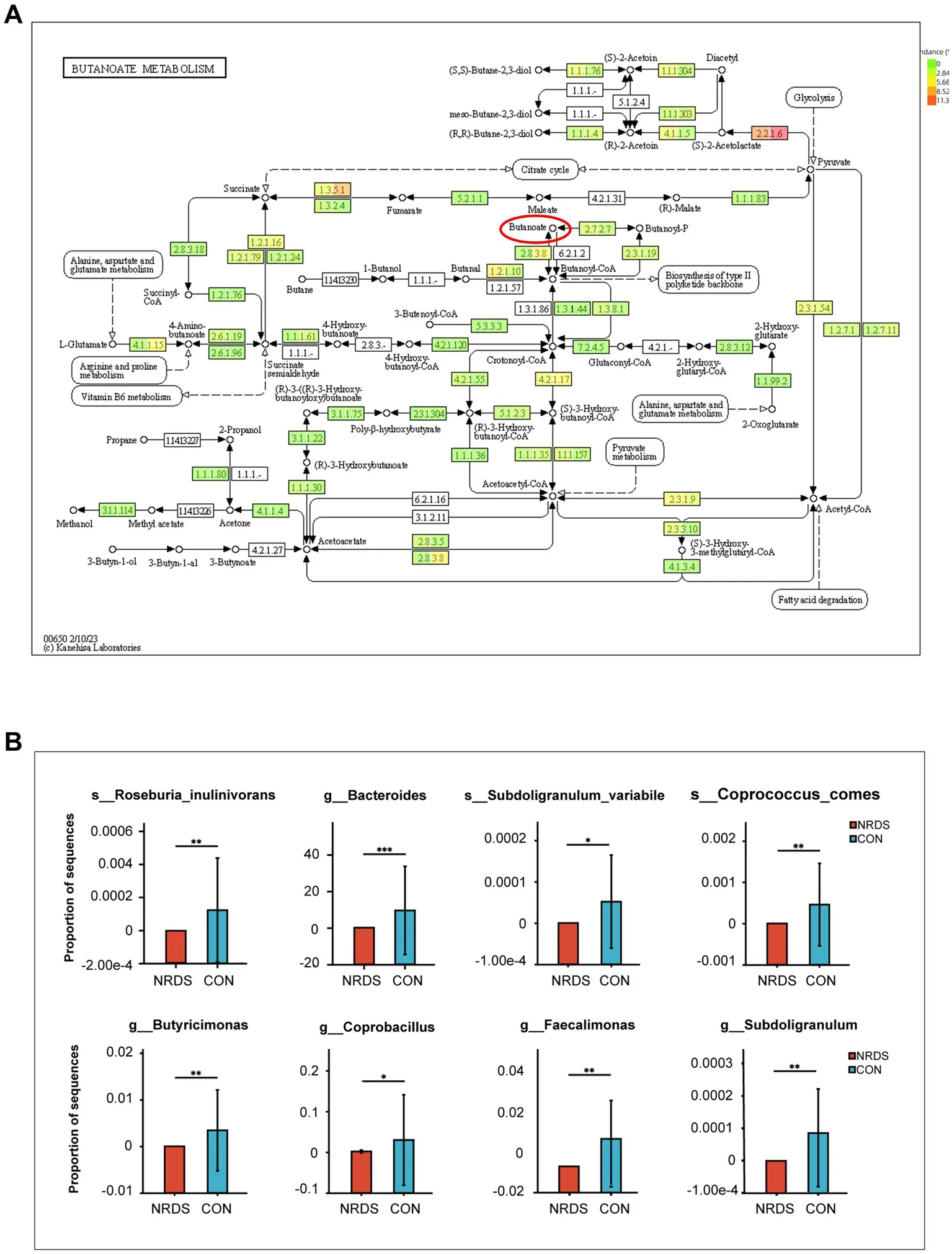
Butyrate biosynthesis impairment in NRDS gut microbiota. **(A)** KEGG pathway map00650 showing key enzyme abundance changes (green: decrease; red: increase). **(B)** Differential enrichment of butyrate-producing microbiota (*p ≤ 0.05, **p ≤ 0.01, ***p ≤ 0.001).

Short-chain fatty acids (SCFAs), which mainly consist of acetate, propionate, and butyrate, serve as the key metabolites produced through the fermentation of dietary fiber by bacteria present in the gastrointestinal tract (Zhang et al., 2023). Butyrate serves a vital function as the primary energy source for epithelial cells in the colon and is recognized as one of the key anti-inflammatory metabolites present in the intestine (Samuelson et al., 2015). Metagenomic analysis revealed compromised butyrate biosynthesis (KEGG map00650) in NRDS neonates. Key enzymes including butyryl-CoA dehydrogenase and acetate-CoA transferase showed reduced abundance compared to CON group (Figure 4A). Consistently, butyrate-producing taxa including *Bacteroides, Roseburia inulinivorans*, and *Butyricicoccus* were depleted in NRDS (Figure 4B). These findings indicate differences in the microbial functional potential related to butyrate synthesis in the NRDS group; however, their relationship with gut barrier function and systemic inflammation cannot be determined from the present cross-sectional data.

#### 3.4.2 Contribution analysis of species and functions

**Figure 5:**
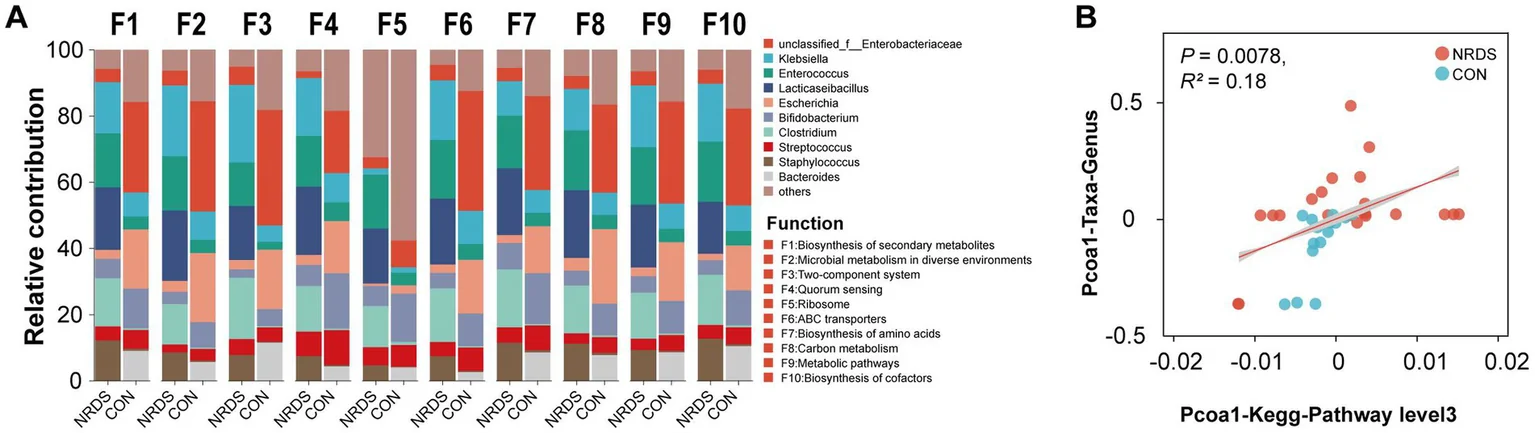
Analysis of species contribution to functionality. **(A)** Analysis of relative contribution of different taxa. **(B)** Regression analysis of species (genus level) and function.

At KEGG Level 3, the analysis of species and functional contributions indicated that the top 10 species and KOs in terms of total abundance in both the NRDS and the CON groups primarily involved F1-F10. The functional contributions of *Klebsiella, Enterococcus, Clostridium,* and *Staphylococcus* were significantly higher in the NRDS group compared to the CON group. Conversely, the functional contributions of *Escherichia coli*, *Bifidobacterium*, and *Bacteroides* were significantly lower in the NRDS group than in the CON group (Figure 5A). Linear regression revealed moderate but significant correlation between genus-level composition and functional profiles (*R^2^* = 0.164, *p* = 0.011), indicating an association between microbial community composition and functional profiles in the NRDS group (Figure 5B).

### 3.5 Relationship of gut microbiota with clinical factors

**Figure 6:**
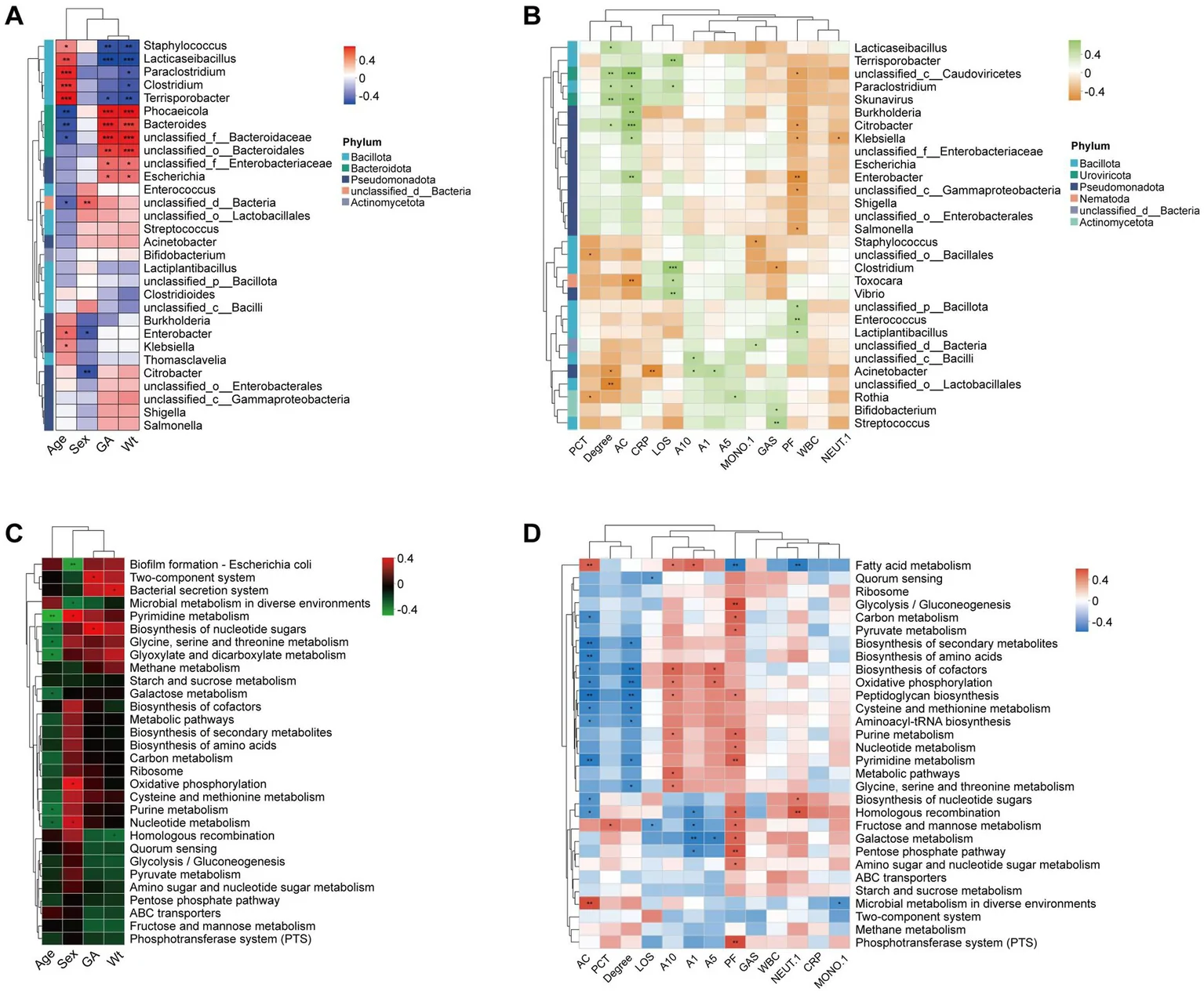
Microbial and functional correlations with clinical parameters. **(A)** Clinical parameters and species correlations in the NRDS and CON groups. **(B)** Clinical parameters and species correlations in the NRDS group. **(C)** Clinical indicators and functional correlations in the NRDS and CON groups. **(D)** Clinical indicators and functional correlations in the NRDS groups. GA, Gestational age; Wt, Birth weight; red, light green: positive correlation; blue, orange, dark green: negative correlation (*p ≤ 0.05, **p ≤ 0.01, ***p ≤ 0.001).

Through the analysis of the correlation between dominant species and clinical indicators, we found that both the NRDS group and the CON group exhibited a significant positive correlation between age and five candidate microorganisms: *Paraclostridium*, *Clostridium*, *Terrisporobacter*, *Lactobacillus rhamnosus*, and *Staphylococcus* (Figure 6A). In the NRDS group, abnormal inflammatory indicators (PCT, CRP, MONO.1, WBC) were found to be negatively correlated with *Staphylococcus* and *Acinetobacter*. The severity of NRDS showed significant positive correlations with three candidate microorganisms: *Clostridium*, *Skunavirus*, and Citrobacter, while a significant negative correlation was observed with *Acinetobacter*. Length of hospital stay (LOS) was positively correlated with *Terrisporobacter*, *Clostridium*, and *Vibrio*. Apgar scores (A1/A5/A10) demonstrated significant positive correlations with *Acinetobacter*. The gestational age score (GAS) exhibited significant positive correlations with *Bifidobacterium* and *Streptococcus*, alongside a significant negative correlation with *Clostridium* (Figure 6B). Further analysis of the correlation between KEGG pathways and clinical indicators revealed that in both the NRDS and CON groups, postnatal age was significantly negatively correlated with galactose metabolism (Figure 6C). In the NRDS group, Apgar scores were significantly positively correlated with the biosynthesis of cofactors and fatty acid metabolism, while they were significantly negatively correlated with galactose metabolism. Abnormal inflammatory indicators were significantly positively correlated with the biosynthesis of nucleotide sugars and homologous recombination, and significantly negatively correlated with fatty acid metabolism (Figure 6D).

### 3.6 Diagnostic potential of gut microbiota in NRDS

**Figure 7:**
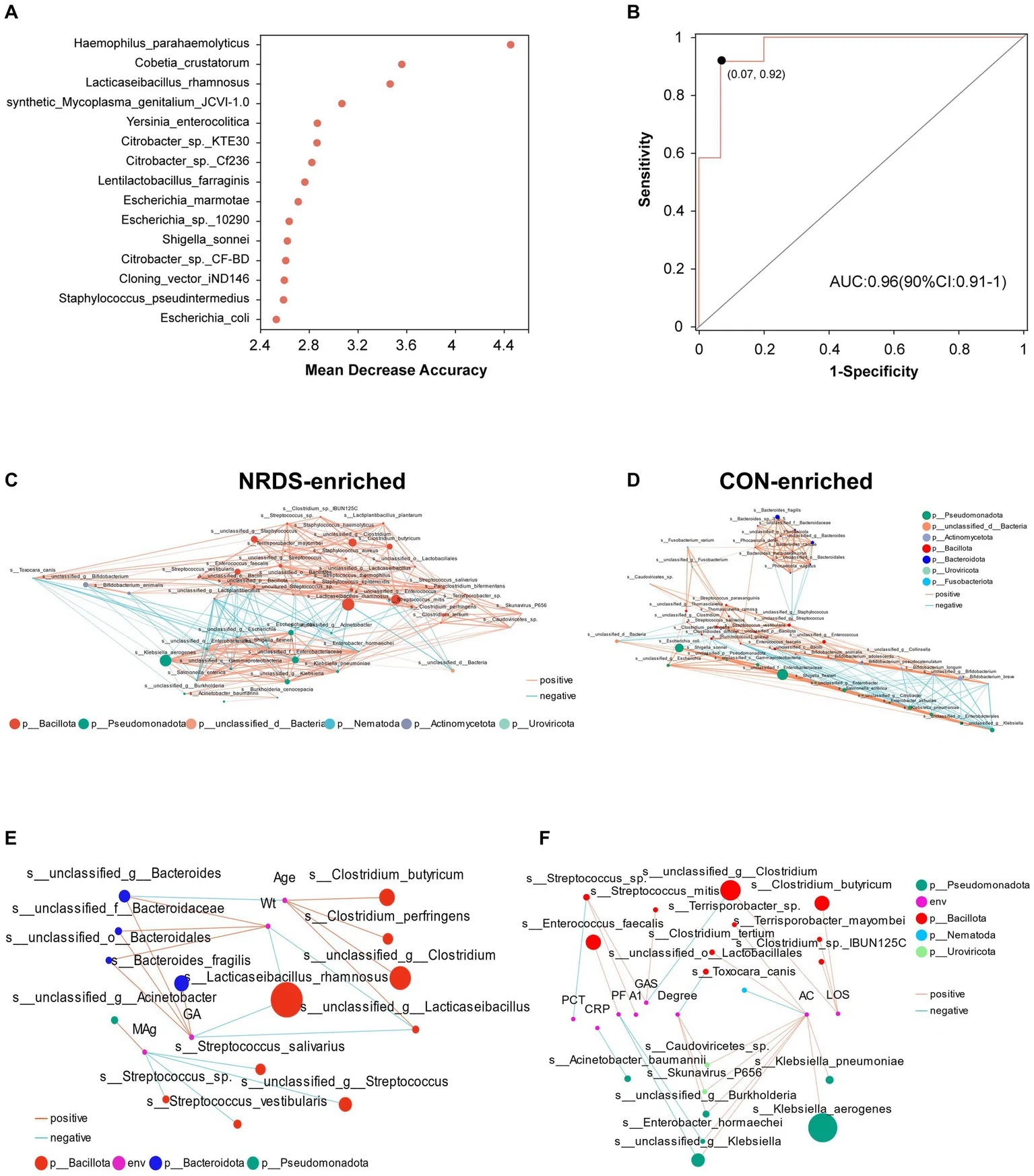
Diagnostic model construction and network analysis. **(A)** Species importance ranking chart. **(B)** ROC curve of the random forest classifier using the top 10 species biomarkers. AUC = 0.96 (90% CI, 0.91–1.00). The optimal cutoff yields sensitivity = 0.92 and 1-specificity = 0.07. ROC, Receiver operating characteristic curve; CI, Confidence interval. **(C,D)** Species correlation network analysis. **(E,F)** Clinical-microbial correlations: Cross-group **(E)** and NRDS-specific **(F)**. Red line: Positive correlation, blue line: Negative correlation; the larger the node, the higher the abundance of the species, and the more lines, the closer the correlation relationship, ENV: Clinical indicators. WT, Birth weight; MAg, Maternal age; PF, Porcine lung phospholipid; AC, Antenatal corticosteroids.

A random forest classifier was constructed and validated using the area under the curve (AUC), identifying the top 15 most important microbes (Figure 7A). Among the top 10 species biomarkers identified, the AUC reached 96% (90% CI, 0.91–1.00) (Figure 7B); at the optimal cutoff (1-specificity = 0.07), the sensitivity was 0.92. However, this result was obtained within a single cohort of modest sample size without independent external validation; it should therefore be interpreted as preliminary and hypothesis-generating, and validation in a larger independent cohort remains necessary. Species correlation analysis revealed that the phylum Nematoda was exclusively enriched in NRDS patients, while the Bacteroidota and Fusobacteriota were uniquely enriched in the CON group. Notably, the phylum Uroviricota demonstrated a positive correlation with Pseudomonadota in the NRDS group, with no correlation observed in the CON group (Figures 7C,D). Furthermore, analysis of the correlation between clinical indicators and species indicated that in both groups, body weight and gestational age were negatively correlated with Bacillota, while positively correlated with Pseudomonadota and Bacteroidota (Figure 7E). In the NRDS group, the LOS was positively correlated with Pseudomonas (Figure 7F).

## 4 Discussion

Research indicates that the occurrence of various pulmonary diseases is associated with intestinal dysbiosis (Amabebe et al., 2020). For instance, in children with asthma, the overall structure of the gut microbiota remains stable during the early stages of the disease; however, the proportions of specific bacterial genera undergo significant changes, characterized by an increase in the abundance of Bacteroides and a decrease in Roseburia. Probiotic interventions can effectively modulate the imbalance of gut microbiota and reduce pulmonary inflammatory responses (Puccetti et al., 2022). In individuals with chronic obstructive pulmonary disease (COPD), the relative abundance of *Streptococcus* increases, whereas the relative abundance of *Bacteroides* decreases (Collaco et al., 2022). Furthermore, supplementation with *Bifidobacterium breve* and *Lactobacillus rhamnosus* has been found to mitigate lung lesions in a mouse model of COPD (Marchesi, 2010). These changes in gut microbiota are closely linked to lung diseases. Through metagenomic analysis, this study revealed that the structure and function of the gut microbiota in NRDS patients have undergone changes.

Research indicates that the human gut microbiota is predominantly composed of *Bacteroidota* and Bacillota, with additional contributions from *Pseudomonadota* and *Actinomycetota* (Tap et al., 2009). In this study, we observed significant alterations in the structure of the gut microbiota within the NRDS group, indicating that NRDS was associated with differences in early gut microbial colonization patterns. Within a specific range, Bacillota maintain the integrity of the intestinal mucosal barrier by producing SCFAs, thereby mitigating the body’s inflammatory response (Ahlawat et al., 2021). *Pseudomonadota* (formerly *Proteobacteria*) constitutes one of the most prevalent groups in the gut microbiota and plays a crucial role in the regulation of the immune system (Cheng et al., 2020). Previous studies have demonstrated that birth and feeding methods influence the colonization of gut microbiota. The gut microbiota of infants who are born naturally and breastfed predominantly consists of *Bacteroidota* (Zhao et al., 2021). The *Bacteroidota* phylum is recognized for its role in energy metabolism, providing energy to the host while also modulating the host’s immune response through various pathways to maintain ecological balance within the body (Amabebe et al., 2020). A rise in Bacillota alongside a decrease in *Pseudomonadota* and *Bacteroidota* is strongly linked to inflammatory diseases. Importantly, the levels of *Enterococcus* in the feces of individuals suffering from NRDS were markedly increased. This genus stimulates host immune mechanisms and facilitates pathogen tolerance through its peptidoglycan structure and hydrolase functionality (Kim et al., 2019). In conclusion, the composition of gut microbiota is modified in patients with NRDS, and additional research is necessary to understand its effects on lung disease.

This research revealed that the gut microbiota in children with NRDS displayed not only changes in composition but also significant functional variations. Specifically, the enrichment of metabolic pathways beneficial to health was diminished in the NRDS group, whereas the enrichment of pathways associated with disease promotion was heightened. Furthermore, the enrichment of pathways related to biodegradation and metabolism increased, whereas the enrichment of biosynthetic pathways decreased. Within the host, the components of intestinal microbiota and their metabolites greatly affect inflammatory and immune reactions, both locally and systemically. Commensal microorganisms in the intestine, such as *Bifidobacterium* and *Bacteroidota*, are known to induce the production of antimicrobial peptides, secretory immunoglobulin A, and proinflammatory cytokines (Tap et al., 2009). Importantly, microbial metabolites, particularly SCFAs, can directly or indirectly modulate various immune cells, thereby influencing inflammatory responses (Lin et al., 2022). The primary SCFAs that play crucial roles in regulating immunity, apoptosis, inflammation, and lipid metabolism include acetate, propionate, and butyrate (Zhang et al., 2023). In this study, we observed a significant reduction in the enrichment of KEGG pathways associated with butyrate metabolism in the NRDS group. Additionally, the abundance of butyrate-producing microorganisms markedly decreased. Furthermore, the PTS pathway in the NRDS group was enriched. In pathogenic bacterial species, the PTS pathway has been shown to influence the expression of virulence genes [46], and it was found to be enriched in the NRDS group in this study. Consequently, we speculate that the PTS may enhance the pathogenicity of related gut microbiota by increasing the expression of virulence genes, thereby exacerbating the destruction of the intestinal barrier in patients with NRDS. Further linear regression analysis showed that changes in microbial community composition were related to changes in overall functional composition.

The “gut-lung axis” serves as a crucial mechanism through which gut microbiota influence lung diseases (Wypych et al., 2019). Factors such as the mode of birth, feeding practices, and environmental conditions play significant roles in shaping the intestinal microbiota of newborns. This microbiota typically exhibits low diversity, simplistic functions, rapid fluctuations, and substantial individual variation (Enav et al., 2022). This study identified synergistic and mutually exclusive relationships among microorganisms as well as between microorganisms and clinical factors. Previous studies have demonstrated that healthy gut microbiota can significantly increase the production of PS to a certain extent (Li et al., 2024). Most children with NRDS present with PS deficiency at birth, resulting in progressive dyspnea and hypoxia, which impacts microbial colonization of the distal intestine. Furthermore, the gut microbiota may exert immunomodulatory effects on the lungs by influencing gut-derived hormones (Lin et al., 2022). However, it remains challenging to ascertain whether alterations in the gut microbiota are a cause or a consequence of the inflammatory response in children with NRDS. These two factors may interact, functioning either simultaneously or sequentially at various stages of the disease. Nevertheless, the association between inflammatory responses and imbalances in gut microbiota is evident. In recent years, there has been a growing emphasis on “bacterial therapy,” particularly the use of saliva-derived *Streptococcus* 24SMB and oral *Streptococcus* 89a, in the clinical management of respiratory illnesses (Bidossi et al., 2018). Whether NRDS-associated microbial features could serve as therapeutic targets requires investigation in longitudinal and experimental studies.

This study has several limitations. Its cross-sectional design and single-time-point stool sampling preclude causal and temporal inferences, and the observed microbial differences may be associated with NRDS or other perinatal factors. The lack of bronchoalveolar lavage samples prevented direct evaluation of lung microbiota–immune interactions. In addition, the small sample size limits the robustness and generalizability of the taxonomic, functional, and classification findings. Further validation in larger, independent, multicenter longitudinal cohorts with serial sampling and appropriately matched controls is warranted.

## 5 Conclusion

NRDS was associated with differences in gut microbial composition and shotgun-metagenomic functional profiles compared with the control group. Because of the cross-sectional design, these findings do not establish whether the observed microbial alterations preceded or resulted from NRDS. Larger longitudinal studies with serial sampling and appropriately matched controls are required to clarify their temporal relationship and clinical relevance.

## Data Availability

The datasets generated and analysed during the current study are available in the NCBI SRA repository (PRJNA1231247, https://www.ncbi.nlm.nih.gov/bioproject/PRJNA1231247). De-identified clinical metadata are available from the corresponding author upon reasonable request. The data release was approved by the Biomedical Research Ethics Committee of the Second Affiliated Hospital of Nanchang University (Approval No. O - Medical Research Ethics Review [2024] No. (90)).

## Footnotes

1
www.illumina.com

2
https://www.majorbio.com/
